# The Manse

**Published:** 1862-04

**Authors:** 


### THE MANSE

is another name for the "Parsonage," and is understood to mean a dwelling-house belonging to a church, congregation, or society, used as a residence for its minister. The advantages of such an arrangement are numerous and important, both to the clergyman and to his people.

1st. No time need be lost, when getting a new minister, in hunting a suitable house for his family. THE MANSE BEING PAID FOR.

2d. The pastor would be always sure of a shelter and a home, whatever might be the scarcity of houses, or however great the pecuniary inflation; he would be also exempt from any inconvenience resulting from the change of landlords, from their death, from their failure, or from their impositions and exactions; while his people would have a personal and pecuniary interest in keeping it always in full repair, which would be done, in most instances, without the direct expenditure of money, and thus not be felt; as almost every society would number among its members the carpenter, the painter, the mason, the plasterer, etc., etc. In this way the manse system obviates one of the chief items of expenditure, and removes one of the greatest sources of annoyance.

3d. No man of culture, intelligence, and liberality, and especially no Bible Christian, will fail to feel in reference to his minister, that "the laborer is worthy of his hire;" and it is quite as evident, that the less a society *feels* the tax for supporting a preached Gospel, the better. Thoughtful men also know, that a large majority of the clergy of all religious denominations, are inadequately paid. In no department of business-life, is the pecuniary compensation so small, so disproportioned to the talent, the capacity, the mental power, and the moral worth, as in reference to ministers of the Gospel. The same intelligence, the same probity, the same industry, the same conscientiousness, would not in any other direction fail to realize a multifold greater compensation, as far as mere money is concerned. If, then, every society had its manse, the chief item of the minister's expenditure would be at once canceled, and thus his adequate support would be less likely to be felt as a burden; there would also be less necessity for dependence on the contributions of those who were not members of the church; and a greater feeling of independence on the part of the minister in the discharge of his duty as a faithful and fearless servant of Christ, would inevitably ensue.

4th. If the manse were paid for, it would be equivalent to an increase of perhaps twenty-five per cent to the minister's salary, in the great majority of cases; for the "house-rent," costing the people nothing, in the present, would gradually be lost sight of as an item, while the "old figure" for the minister's support would be the sum which the people would feel ought to be raised; thus, the house-rent thrown in, would inure to the benefit of the whole congregation in a greatly increased degree; in that the minister would have more time to devote to the members of his charge; his mind would be less diverted from his great and more appropriate work, by the uncongenial pressure of worldly matters; by the chilling study of how to meet necessary expenditures; by devising annoying and perplexing and humiliating expedients and make-shifts; and by the hard necessity of turning a deaf ear, and a cold eye, and a heartless denial, upon the mendicant, the fallen and the unfortunate at the door, while at the same instant of time, he was penning in his study an appeal to his people for the habitual exercise of godlike charity.

That society is greatly and unwisely derelict, which, in its pastor's salary, allows no margin for benevolent deeds; because not only does that heart grow warmer and better and more divine as it practices good-doing to the desolate, but it infuses its spirit into every page written, into every sermon spoken, and into every prayer uttered, to kindle up other hearts, to warm up other benevolences, and thus wake a whole community to godlike practices; and never since the world began did any such community ever fail of a largely-increased worldly thrift, to say nothing of the promise of the life that is to come; because he who "considereth the poor, lendeth to the Lord," who promises to repay with a heaping measure, running over in this life, and in the world to come, life everlasting.

5th. The amount paid by clergymen for rent, in any one year, as to the larger denominations, will run from fifty to eighty thousand dollars, which, as it were, goes out of the family, out of the church, and hence is equivalent to that much absolutely sunk; it is that much paid annually as interest, and is that much lost to the great fund for the promotion of the Redeemer's kingdom, and must be thought of with regret by every practical, thinking, working Christian.

6th. This is but the half. The ultimate working of the manse system, would, without taking up space to write out the argument, be to these two ends.

(1st.) The salaries of the clergy would eventually average what they do now, with the rent thrown in.

(2d.) This fifty to eighty thousand dollars a year would not only be that much saved, but an equal amount would be paid in addition; for the minister would not only not have to pay out a hundred dollars for rent, but would have his rent thrown in; thus it would be equivalent to two hundred dollars to him; to a hundred, or a hundred and sixty thousand dollars a year to any large denomination.

7th. If the manse scheme were proposed to-morrow, the people would at once begin to think and talk about it; and inevitably a growing interest would be felt in it, in every society where there were any true piety and zeal for the cause of Christ; and gradually, sums smaller or larger, would be left by will by the members; for it would be something tangible; something permanent; something connected with the material interest of the community, to say nothing of the more important spiritual benefits, and the mothers in Israel and the patriarchs in the church, and the Dorcases of each society, would, as they passed away, leave tokens of remembrance in this direction, so that eventually the price of every manse would, in reality, be that much saved to the church, since but for the manse, it would have gone into other channels.

8th. Further: in villages and small towns, half an acre of ground, or one or two or three acres, would very naturally surround the manse as a part of it, and in the country five or ten acres. Benevolent persons would say in life, or at death: "I will give this lot of ground for a manse, if the people will build the house." In many cases this house could be erected by the mechanics, lumber and hardware merchants of the place, without their "feeling it;" and thus a generous emulation in liberality would be engendered, without the people feeling that they were giving their money directly to the minister; in fact, they would be only improving their own property, with benefits, pecuniary and otherwise, resulting directly to themselves, to their children, and their children's children; and with a bit of land attached to each manse, each conscientious clergyman would feel it to be his pleasure and interest, as well as his duty to do somewhat for its improvement and embellishment; and in time, the manse would, by reason of the intelligence and taste necessarily belonging to cultivated minds, become the beauty and the pride of the community round about; and the young of the congregation or parish would have associations connected with their minister of the sweetest character; associations of green trees, and flowers; of bowers and of graveled walks; of the dew-drops and the singing-birds; of the early morning, with the sunshine and the cloud of showery April; the perfumes of leafy June, as well as of the sweet sadness of autumn; and through it all would run the more hallowed associations of the pastor of one's youth; his unvarying smiles of welcome; his sympathizing tear at the funeral; that merry twinkle of the eye which comes from heart-gladness at the wedding; and the tremulous utterances from the sacred desk, which well up from a heart in deep concern for the soul's best interest of those who are listeners to the preached word. Each of these will leave an impress on the mind of childhood; together, they would make it so vivid that its memories would not fade from the heart or the affections to the latest hour of the longest life, while they all would have a tranquillizing, a soothing, a restraining and a sanctifying influence even, of no small importance. Contrast this with the clergyman living in some unrepaired, dilapidated dwelling, or on some bald situation where not a tree or bush is to be seen, with that stereotype sadness which soon enshrouds the face which answers to a mind habitually disturbed by painful economies, by pressing pecuniary obligations or scanty payments, long past due, and can a child fail to attach "*désagréments*" to the religion which that minister professes, and thus be unfavorably affected towards it? There is much, very much, in this thought which may be profitably matured in any Christian mind.

9th. Another benefit is, that on a very small piece of land, intelligent industry may raise enough to meet a considerable portion of a minister's expenditure, and thus, as his family grows larger, his salary remaining the same, he may have some resource for this increased cost of living, and thus avoid that wearing harassment which attends the inquiries: How am I to educate my children; how am I to meet the greater cost of dressing them as they grow older?

10th. In weak congregations, whether from their just struggling into existence, or from emigration, deaths, failures, monetary crises, colonization, or the like, the manse, with a small amount of land, would be something to fall back upon; for by cultivating it more rigidly, money would, as it were, be created; it would be a "mine" of considerable value, and whether much or little, it could never be exhausted. From a single acre of good soil, scientific cultivation will cause an annual yield of from one to two hundred dollars; and with no house-rent to pay, this would go very far toward supporting a family in very many parts of the country; at least, it would be the means of keeping up the stated ordinances of God's house, without intermission, until better times came.

11th. The minister could make more out of a small piece of land, with less outlay of money, than almost any other person, because he could do it without hired help; without horse, wagon, plow, or seed; for one member of his parish could plow half a day for him; another could chop wood half a day; another haul half a day; another furnish a little seed of one kind; another of another kind, and so on; and all these "without missing it," while the very fact of the kindness done, (too small to make an obligation of it, as against the minister, yet large enough to draw out his kindly feelings,) does but afford the giver an opportunity of a good turn without cost, to the one who, of all the persons of his acquaintance, has the most claims upon him; and toward whom a good turn done, gives the most unalloyed satisfaction. And if the minister's wife had a little of any thing to sell, of a surplus, or which she might think she could not so well afford to use herself, it may reasonably be supposed that almost any member of the society would prefer the purchase, and even embrace the opportunity of giving a higher price, as a means of a little donation, without its being felt as such. That such interchanges as these, in connection with the manse scheme, would have a happy social influence, in every bearing, will scarcely be denied.

12th. Last, not least, with a little land to cultivate as a means of that exercise, without which vigorous health is an absolute impossibility, the manse scheme is of incalculable value. On a single acre of land, a man can expend two hours a day, for every day in the year, in which the ground is not frozen, or there is no rain; this would save the expense of a horse to ride for exercise; or that most intolerable of all tasks, to an educated, active mind, an aimless, monotonous walk of a mile or two, and back. To be sure, a walk or ride is better than nothing; but the same amount of time spent in doing something which is profitable, interesting, and agreeable, is not only of treble value as regards its healthful influence, but it is that much time saved to the man, to his people, and to the world; for that hour has not only secured a variety of healthful influences, but it is an hour saved, and there is the result in work to show for it.

The want of facilities for exercise, is the great trouble with clergymen. The manse scheme not only gives them exercise, except in cities and large towns, but gives them remunerative exercise; and gives more time for study, by relieving them from pecuniary pressure; and also by increased health, enables them to study to greater advantage in the same space of time; thus in its reflex influences again blessing the giver, the church to which he belongs, the community in which he lives, and society at large. In view of the whole subject, what lover of the Lord Redeemer is there, who might not do the Church a large service by determining to take the initiative in founding a manse for his church, which shall be an enduring source of pecuniary and spiritual good to the congregation long after the Master has called him to go up higher, and thus have his work to "follow" him, till time shall be no more?

